# Atlanta Medical College

**Published:** 1856-06

**Authors:** 


### EDITORIAL AND MISCELLANEOUS.

### ATLANTA MEDICAL COLLEGE.

The second course of Lectures commenced in this institution on the 1st day of May, with a brilliant Introductory Address from Alexander Means, M. D., Professor of Chemistry and Pharmacy. We had hoped to have been able to present this address to our readers, in the present number of the Journal, but have found it to be impracticable; we are assured, however, that it will be presented in another form at an early day, when each of our subscribers will be furnished with a copy of this truly eloquent address.

In the highest order of oratory, (that based upon science and religion,) we have no hesitation in asserting, that Prof. Means has no superior in this Union; and though at all times, upon occasions like this, of a public character, chaining his audience with an intense interest, he must be heard, to be fully appreciated; by the man of *science*, and in the *place of science*, (the chemical lecture room) surrounded by the wonderful modern appliances for illustrating chemical laws; where, under the exciting influences of the subject and a consciousness of power, his audience almost *see* into the lowest depths of the volcano, and are made to comprehend the phenomena connected with the belching forth of fiery lava; or again are carried into the storm cloud, and as it were, catching illumination from the lightning's flash, are allowed to behold, in close proximity, in all their beauty and grandeur, the wonders of the heavens. The institution has recently been furnished with a magnificent chemical apparatus, and other valuable appliances connected with the various departments; and upon the testimony of those who have had opportunities of comparison, by attendance upon lectures in other institutions, we hesitate not to say, that medical science is here taught in an *eminently practical* manner—the wants of the student, when he enters upon the active pursuit of his profession, are looked to, we believe, with *peculiar* interest by every member of the faculty—and we are happy to know that our efforts are fully appreciated by those who have honored us with their presence, and are sustaining and encouraging us, by their gentlemanly demeanor and devotion to duty. The truth is, and we do not know that there is any impropriety in saying it, we have a working establishment—both faculty and students—and with the motto of *progress* inscribed upon our banners, we mean to go on to deserve and build up a high position.

The number of matriculates is already larger than that of the previous session at its *close*, and the class in regular attendance presents a large excess over that of the last course, with almost daily accessions; which we think fully justifies us in the assertion, that we shall close up this term with a proportion of increase not less than from one-third to one-half.

In reference to the character of those who compose the class, we can say, that it is all that we could desire, and fully equal (if not superior) in intelligence to the average medical classes of the most prominent institutions of the land, and in reference to the energy with which they are devoting themselves to the acquisition of knowledge, we think *far superior;* which we account for, to a considerable extent, upon the principle that the most of those who come here, are looking more to the prosecution of their studies continuously and perseveringly, than to the attractions of idleness and leisure, so apt to prevail during the summer season, with those who are merely pursuing a prescribed *routine*, and who are relying more upon *time*, than application, to confer upon them the degree.

In conclusion of this hasty article we would say, that our halls are open to the profession from every quarter, and we invite the fullest scrutiny and investigation, and it will at all times give us pleasure to afford any true medical man an opportunity of profit—if it may be—or of comparing our works and professions.

